# *Michaelmoelleria* (Gesneriaceae), a new lithophilous dwelling genus and species with zigzag corolla tube from southern Vietnam

**DOI:** 10.3897/phytokeys.146.49731

**Published:** 2020-05-08

**Authors:** Fang Wen, Zi-Bing Xin, Long-Fei Fu, Shu Li, Lan-Ying Su, Stephen Maciejewski, Zhang-Jie Huang, Truong Van Do, Yi-Gang Wei

**Affiliations:** 1 Guangxi Key Laboratory of Plant Conservation and Restoration Ecology in Karst Terrain, Guilin Botanical Garden, Guangxi Institute of Botany, Guangxi Zhuangzu Autonomous Region and Chinese Academy of Sciences, Guilin 541006, China; 2 Gesneriad Conservation Center of China (GCCC), Guilin Botanical Garden, Chinese Academy of Sciences, Guilin 541006, China; 3 Key Laboratory of Plant Resources Conservation and Sustainable Utilization, South China Botanical Garden, Chinese Academy of Sciences, Guangzhou 510650, China; 4 The Gesneriad Society, 2030 Fitzwater Street, Philadelphia, PA 19146, USA; 5 Vietnam National Museum of Nature, Vietnam Academy of Science & Technology, 18 Hoang Quoc Viet, Hanoi, Vietnam; 6 Graduate University of Science and Technology, Vietnam Academy of Science & Technology, 18 Hoang Quoc Viet, Hanoi, Vietnam

**Keywords:** *
Cathayanthe
*, cliff-dwelling, *
Deinostigma
*, Didymocarpoideae, flora of Vietnam, IUCN, phylogeny, *
Tribounia
*

## Abstract

*Michaelmoelleria*, a new genus from southern Vietnam is described with a single species, *M. vietnamensis*. The new genus is morphologically most similar to *Deinostigma* and *Tribounia* but it differs from the latter two by having four fertile stamens. Nuclear ribosomal internal transcribed spacer (ITS) region and plastid *trnL-F* intron spacer (*trnL-F*) DNA sequence data from the new genus and eighty-seven species representing 42 genera within tribe Didymocarpeae are used to resolve its generic placement. The molecular evidence reveals that it is most closely related to *Cathayanthe* rather than *Deinostigma* and *Tribounia*. The chromosome number is counted as *2n* = *36* that further clarified its distinction comparing to the related genera within tribe Didymocarpeae. A global conservation assessment is also performed and classifies *Michaelmoelleria vietnamensis* as Critically Endangered (CR).

## Introduction

The generic delimitations in Asian Gesneriaceae are often ambiguous due to significant overlap in characters between genera (Burtt 1977), which has led to many revisions including synonymization of small and monotypic genera (Möller et al. 2011a, b; Puglisi et al. 2011; Weber et al. 2011a, b; Möller et al. 2016) and establishment or resurrection of new or previously synonymized genera in recent years (Wei et al. 2010; Weber et al. 2011c; Middleton and Triboun 2012; Middleton and Möller 2012; Middleton et al. 2014, 2015, 2018). The combined phylogenetic-morphological approach was therefore performed as it takes both molecular and morphological evidence into account (Wei et al. 2010).

Vietnam comprises 331,000 km^2^ situated on the eastern Indochinese Peninsula. The flora of Vietnam contains ca. 12,000 vascular plant species (Averyanov et al. 2003). Despite the high species diversity, it is still under-sampled, with only 43 collections per 100 km^2^ (Middleton et al. 2019). Due to the status of uneven collection among the countries within Southeast Asia, Thailand, for example, has an estimate of 75 collections per 100 km^2^ (Middleton et al. 2019) resulting in species richness and endemics of Gesneriaceae being much higher than those in Vietnam. Recently discovered new genera such as *Somrania* D.J.Middleton (Middleton and Triboun 2012), *Tribounia* D.J.Middleton (Middleton and Möller 2012), *Chayamaritia* D.J.Middleton & Mich.Möller (Middleton et al. 2015) and *Rachunia* D.J.Middleton & C.Puglisi (Middleton et al. 2018) further clarified this status. Besides, the uneven collection also seems to occur within Vietnam. Fu et al. (2019) reported no record of *Elatostema* from Vietnam’s southernmost provinces and partially ascribed this pattern to unequal sampling effort across the country. Therefore, they proposed a more significant sampling effort in southern Vietnam.

**Figure 1.**
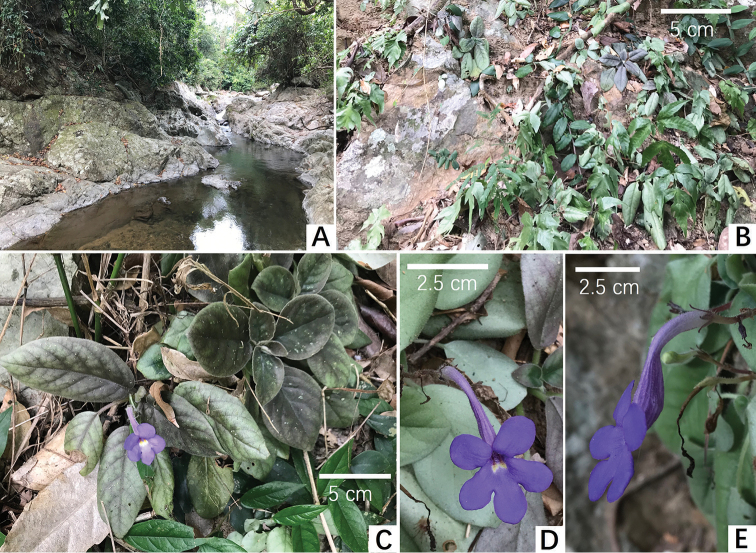
Photos of the new genus, *Michaelmoelleria*, the population in natural habitat. *M. vietnamensis* F.Wen, Z.B.Xin & T.V.Do **A** habitat along the La stream **B** habit **C** plants in flower **D** frontal view of flower in natural habitat **E** lateral view of flower in natural habitat. Photos by Fang Wen, arranged by Wen-Hua Xu.

As part of ongoing research into the diversity of Gesneriaceae in Vietnam, a group of botanists from the Vietnam National Museum of Nature (VNMN) and the Gesneriad Conservation Center of China (GCCC) undertook an extensive fieldtrip in southern Vietnam in 2018. A plant belonging to Gesneriaceae was collected in La stream, Tay Giang community, Tay Son district, Binh Dinh province, close to the belt of An Khe city, Gia Lai province, southern Vietnam (Fig. 1). Surprisingly, this unknown species was unable to be placed in any genus based on morphological characters. Superficially, it shares some similarities to *Deinostigma* and *Tribounia*, but would be easily distinguished from the former by its distinct characters of the long and zigzag corolla tube and rounded corolla lobes and from the latter by its four stamens and a 2-lobed stigma. To confirm the generic placement of this species, molecular and cytological experiments were also performed. After consulting the relevant literature (Wang et al. 1998; Ho 2000; Li and Wang 2005; Phuong 2005; Wei et al. 2010; Wen et al. 2019) along with the molecular and cytological evidence, we concluded that this new species was assignable to a new genus, *Michaelmoelleria* gen. nov. This new genus will be an addition to the ongoing project of ‘Flora of Vietnam’ and ‘Flora of Cambodia, Laos and Vietnam’.

## Materials and methods

### Ethics statement

The locations where this new species was collected were not in any protected area. No specific permissions were required to enter these areas. Our field studies did not involve any endangered or protected species. Special permits to conduct this research were not required.

### Material collection

This new species/genus has been monitored in the field by staff from the VNMN and grown by the authors at the nursery of the VNMN and the GCCC since the plants were collected. We also collected leaf materials of this proposed new species, using silica gel to dry them in the field for DNA extraction.

### Morphological observations and specimens examined

A study of the new genus *Michaelmoelleria* and the only currently known species, *M. vietnamensis*, from southern Vietnam, was undertaken. All available specimens of *Michaelmoelleria* are stored in the following herbaria in China and Vietnam: IBK and VNMN (herbarium acronyms according to Index Herbariorum; Thiers 2016, 2019). All morphological characters were studied using a dissecting microscope (SZX16, Olympus, Tokyo, Japan). Characteristics were described using the applicable terminology presented by Wang et al. (1998). The morphological comparison with other species was based on the study of living plants in the field, in cultivation in the VNMN and the GCCC, and herbarium specimens.

### Genomic DNA extraction, PCR amplification, and Sequencing

To confirm the placement of this new plant, we performed phylogenetic inference of DNA sequence data obtained from the nuclear ribosomal internal transcribed spacer (ITS) region and the plastid *trnL-F* intron spacer (*trnL-F*). Eighty-seven species representing 42 genera as in-group and two species representing one genus as an out-group, including nearly all genera within tribe Didymocarpeae, were sampled. DNA extraction, PCR amplification, and sequencing were performed, following Wei et al. (2013). Sequences obtained from this study and GenBank are listed in the Appendix I.

### Phylogenetic analysis

Sequence data were edited and assembled using Lasergene Navigator 7.1 (DNAstar, Madison, Wisconsin, USA). Cleaned sequences were aligned with Geneious R11 (Kearse et al. 2012). Regions of ambiguous alignment and sites with more than 80% missing data were excluded during analyses (Sun et al. 2018). Phylogenetic analyses were conducted using Bayesian inference (BI) and maximum likelihood (ML) methods. ITS and *trnL-F* datasets were used to construct the ML tree independently to evaluate the congruence between two makers. As there were no hard incongruences (Nishii et al. 2015), we performed the following analysis using a combined dataset. Best-fit DNA substitution models were selected using the Akaike Information Criterion (AIC) in Modeltest v 2.7 (Posada and Crandall 1998). Modeltest determined the best models GTR + G + 1 for the combined dataset. BI analyses were based on a Markov chain algorithm implemented in MRBAYES 3.2.6 (Huelsenbeck and Ronquist 2001). Four chains of the Markov chain Monte Carlo (MCMC) simulation were performed for 4,000,000 generations, each with trees sampled every 100 generations. After discarding the first 25% of the trees, the retained ones were used to calculate the node probability (posterior probability). ML analyses with 1000 bootstrap resampling were conducted using an online version of RAxML-HPC2 v8.2.10 (Stamatakis et al. 2008), available at (http://www.phylo.org/index.php/portal/) (Miller et al. 2010) with the gamma model of rate heterogeneity.

### Chromosome preparations

Leaf cuttings yielded new root tips when grown hydroponically for 2–3 weeks. The new root tips were then pretreated with a solution of 0.002 mol·L^-1^ 8-hydroxyquinoline at 13 °C for 4–5 h. After fixation for 24 h by Carnoy solution (3:1 ethanol: acetic acid) at 4 °C, dissociate, stain, and squash methods followed (Jong and Möller 2000, Christie et al. 2012). The chromosome numbers were determined in at least 20 cells from 10 different root tips with well-spread chromosomes in metaphase and captured using a light microscope (Leica DM 2500, camera Leica DFC420).

## Results

### Molecular phylogenetic studies

**Figure 2.**
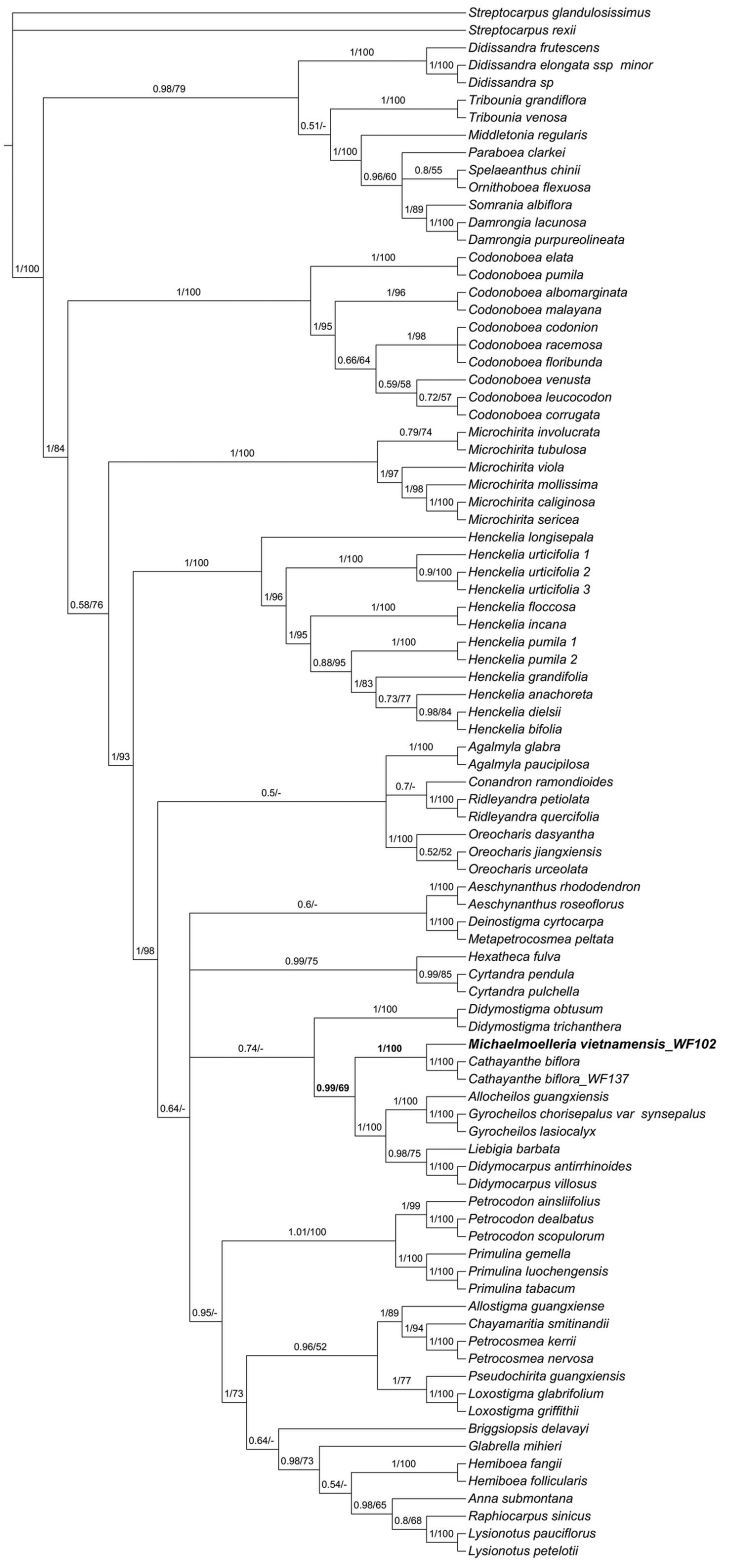
The Bayesian Inference 50% majority rule consensus tree from the combined dataset of *trnL*-*trnF* and ITS. Numbers on the branches indicate the posterior probability (≥0.5) of Bayesian inference analysis and bootstrap values (≥50%) of the maximum likelihood. **BOLDFACE** indicates the new species/new genus. Created by Long-Fei Fu.

The aligned matrix of the combined data (80% missing data were excluded) was 1441 characters, 819 for *trnL-F*, and 622 for ITS. Of the 778 (54.0%) variable characters, 565 (39.2%) were parsimony-informative including indels. BI analysis of the combined dataset resulted in a consensus tree with a well-resolved backbone but included a large polytomy. The BI tree was largely compatible with the best ML tree (Fig. 2).

### Chromosome characteristics

**Figure 3.**
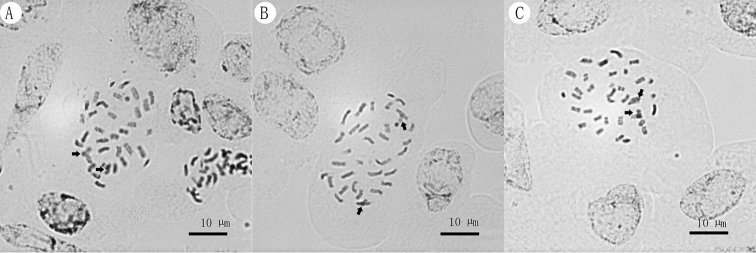
Somatic chromosomes at metaphase of *Michaelmoelleria vietnamensis* F. Wen, Z.B. Xin & T.V. Do, 2*n* = 36 **A–C** are from different cells, solid arrow and, satellites. Photos and arrangements by Lan-Ying Su.

We illustrated the somatic chromosomes of *Michaelmoelleria vietnamensis* at metaphase in Fig. 3. It possesses small chromosomes, falling in the range from 1.51 to 4.15 µm, and we identified the number of the somatic chromosomes as *2n = 36* (Fig. 3), with two relatively small satellites. The chromosomes are small, and the position of centromere could not be determined so that it would not be allowed a detailed karyotype analysis.

### Ecology

Plants of the new taxon were mostly inaccessible, growing on vertical sandstone cliffs and the slope of sandstone hills under tropical evergreen broad-leaved forest. Voucher specimens were made in the usual way (Bridson and Forman 1998) from some accessible plants that could be reached from the base of the cliffs. The conservation assessment was prepared following IUCN (2019).

### Taxonomic treatment

#### Michaelmoelleria

Taxon classificationPlantaeLamialesGesneriaceae

F. Wen, Y.G. Wei & T.V. Do gen. nov.

7D46C9F1-F382-5544-AF9D-5E4A58DBD84C

urn:lsid:ipni.org:names:77209568-1

##### Diagnosis.

*Michaelmoelleria* resembles monotype genus *Cathayanthe*, but differs from the latter by leaf blade glabrous (vs. sericeous to pubescent in *Cathayanthe*, same as order followings); calyx actinomorphic (vs. zygomorphic); fertile stamens 4 (vs. 2), stigma 2, both developed ligulate (vs. 1, subcapitate, divided on 1 side); capsule long linear (vs. fleshly, narrowly ellipsoid). The new genus is also morphologically similar to *Deinostigma* and *Tribounia*, but is easily distinguished from both by having corolla tube narrowly curving to zigzag infundibuliform-tubular, and bent at about 90° angle in the middle of corolla tube (vs. infundibuliform in *Deinostigma*; of a narrow lower tube which widens into an infundibuliform & upper tube which has a prominent boss on the dorsal surface in *Tribounia*, same as order followings), fertile stamens number 4 (vs. 2; 2) and stigma 2-lobed, lobes often gathering together (vs. upper lip usually vestigial and only lower lip developing, broad, flat and weakly 2-lobed; capitate).

##### Type and only known species.

*Michaelmoelleria vietnamensis* F. Wen, Z.B. Xin & T.V. Do, sp. nov.

##### Description.

Herbs, perennial, epipetric, obvious flesh stem, rosette when young and elongated when aging. Leaves basal or clustered at the top of the stem when young but alternate on elongated aerial stem after years of growth; leaf blade ovate to elliptic, glabrous, base cordate to broadly cuneate, apex obtuse. Inflorescences lax, axillary, 1- or 2-flowered cymes; bracts 2. Calyx actinomorphic, 5-parted to the base. Corolla bluish purple to purple, zygomorphic, inside glabrous; tube obviously curved at the middle, dramatically enlarged to be trumpet-shaped from the middle of corolla tube toward limb, much longer than limb; limb 2-lipped; adaxial lip 2-lobed and abaxial lip 3-lobed, lobes rounded to oblate, apex rounded. Stamens 4, included; anthers basifixed, coherent in pairs, thecae divaricate, confluent at apex, dehiscing longitudinally; staminode 1. Disc annular. Ovary narrowly ellipsoid, 1-loculed; placentas 2, parietal, projecting inward and divaricate. Stigma 2, both developed and appressed, lobes ligulate. Capsule straight in relation to pedicel, linear, dehiscing loculicidally to base, splitting along one suture, straight, not twisted.

##### Etymology.

*Michaelmoelleria* was named in honor of Prof./Dr. Michael Möller from the Royal Botanic Garden Edinburgh. He is a well-known botanist studying Old World Gesneriaceae, especially in Africa (Madagascar) and Asia (China), and mentor of the senior author from the 1990s to the present. “*Michaelmoeller*-” (means “Michael Möller”) stands for his full name. “*moeller*” is the English modification of the German family name, “Möller”. Initially, we planned to use “*Moelleria*” as the genus name. However, this name was used in different places three times. They are *Moelleria* Cleve (Bacillariophyta, incertae sedis) [non *Moelleria* Scop. (Spermatophyta, Flacourtiaceae) (≡ *Iroucana* Aubl.)]; [nec *Moelleria* Bres. (Fungi, Clavicipitaceae) (≡ *Moelleriella* Bres.)] [nec *Moelleria* (Freng.) Freng. (Bacillariophyta, Naviculaceae)] (Blanco and Wetzel 2016). Thus, to prevent confusion with those mentioned above, three existing and existed “*Moelleria*”, we consider that using the variant of Dr. Michael Möller’s full name, “*Michaelmoeller*”, to name this new genus to be most appropriate.

##### Distribution and habitat.

Endemic to southern Vietnam, under broadleaved forests in a montane granite area at 140–200 m altitude.

#### Michaelmoelleria vietnamensis

Taxon classificationPlantaeLamialesGesneriaceae

F. Wen, Z.B. Xin & T.V. Do sp. nov.

6FB315EF-7993-5A9C-A78C-A0DC9C766E07

urn:lsid:ipni.org:names:77209569-1

Figs 1 , 4

##### Type.

Vietnam. Binh Dinh province, Tay Son district, Tay Giang community, La stream. 13°55'59"N, 108°45'43"E, ca. 148 m, *WYG180329-01* (holotype: VNMN!, isotypes IBK!).

**Figure 4.**
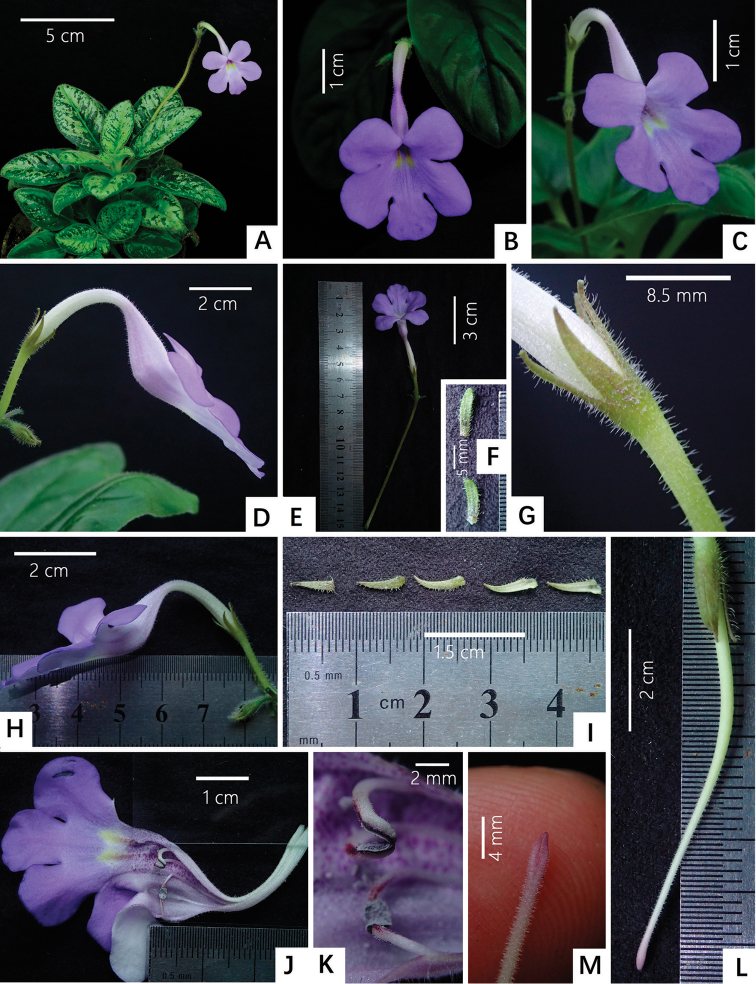
Photos of new genus, *Michaelmoelleria* F. Wen, Y.G. Wei & T.V. Do. *M. vietnamensis* F. Wen, Z.B. Xin & T.V. Do **A** flowering potted plant in GCCC’s greenhouse **B** frontal view of the corolla and the corolla tube **C** different angles of a lateral view of corolla and corolla tube (I) **D** different angles of lateral view of the corolla and the corolla tube (II) **E** cyme **F** bracts (above: adaxial surface; below: abaxial surface) **G** calyx lobes **H** lateral view of flower and bud **I** calyx lobes (the left three: adaxial calyx lobes surfaces; the right two: abaxial calyx lobes surfaces) **J** opened corolla showing stamens and staminode **K** our fertile anthers **L** pistil and calyx **M** stigma. Photos by Fang Wen, arranged by Wen-Hua Xu.

##### Description.

Herbs perennial, rosette when young and stem obviously elongated after years of growth. Stem fleshy, cylindrical, 6–30 cm long, 4–6 mm in diameter, densely white pubescent when young, but glabrescent to glabrous when aging. Leaves alternate on elongated aerial stem, 12–20 cm or more, nearly clustered near the top of the stem and look opposite. Petiole 4–8.5 cm long, 3–3.5 mm in diameter, densely white pubescent. Leaf-blade slightly fleshly to thickly chartaceous, when dried flimsily chartaceous, ovate to elliptic, glabrous, green to dark green, usually with irregular silvery or argenteous spots on the adaxial surface, but silvery-brown to slight yellowish-brown in dry season, 4.5–7 × 2.5–4 cm, base marginally oblique, often slightly cordate, cordate to broadly cuneate, apex obtuse, margin entire, sinuate or with inconspicuously undulate teeth, adaxially and abaxially erectly puberulent; venation alternate along main vein, lateral veins 5–7 on each side of midrib. Cymes axillary near stem apex, fasciculate, 6–10 flowered per plant; peduncle slender, 8–15 cm long, 1–1.5 mm in diameter, brownish-green, densely erectly puberulent; bracts 2, ovate, both usually deflected to same side, 9.8–10.5× 2.5–2.7 mm, adaxially sparsely puberulent, abaxially sparsely puberulent; 1-flowered and 2-flowered per cyme but one of both often abortive; pedicel 1.5–3 cm long, 1–1.2 mm in diameter, green to lime, sparsely extremely white puberulent. Calyx actinomorphic, 5-parted to the base, segments lanceolate to narrowly lanceolate, 8–8.5 mm long, 2–2.3 cm in diameter at the base, apex acute but top usually formed hammer-shape, margin entire, outside sparsely white puberulent, inside glabrous. Corolla obviously curving to zigzag funnelform, zygomorphic, 8–8.5 cm long, outside bluish-purple to purple, densely glandular and glandular-puberulent, inside purple, nearly glabrous, the color of the throat same as the corolla with two brownish-yellow stripes and sparse dark yellow glands on the surfaces of the two stripes. Corolla tube narrowly curving or zigzag infundibuliform-tubular, bent at about 90° angle in the middle of corolla tube, and gradually slightly swollen from the middle to the base of the tube, 3.5–3.9 mm in diameter at middle/corner and 4.8–5.4 mm at the base of tube; dramatically enlarged to be trumpet-shaped from the middle of corolla tube toward limb, 1.9–2.3 cm wide at the orifice of the corolla limb. Corolla limb 2-lipped, adaxial lip 2-lobed, lobes semi-rounded to slightly obliquely oblong-rounded, 1.3–1.5 × 1–1.2 cm; abaxial lip 1.5–1.9 cm long, 3-lobed, middle lobe rounded to oblate and narrowed at the base of middle lobe, 1–1.1 × 0.9–1 cm, lateral lobes orbicular to slightly obliquely oblong-rounded to oblate, 0.9–0.95 × 1.1–1.2 cm. Stamens 4, bigger pair adnate to corolla tube ca. 2.8 cm from the base and smaller pair adnate to corolla tube ca. 2.5 cm from the base, coherent; anthers glabrous; filaments glabrous to very sparsely glandular-puberulent, but near the top of filaments and the part close to anther densely glandular-puberulent, longer pair 8–9 mm long and shorter pair 7–7.5 mm; anthers glabrous, 2.2–2.5 mm long, margin of locule dark purple to purplish-brown; pollen gray; staminode 1, punctate, adnate to corolla tube 2–2.1 cm from base, ca. 1 mm long. Disc annular, ca. 1 mm high, margin entire. Pistil 8–8.5 cm long; ovary cylindric-linear, glabrous, 3.5–4 cm long, pale green; style linear, densely erect glandular and glandular-puberulent, ca. 4.5 cm long; stigmas 2-lobed, often gathering together but slightly opened at the end of flower, lobes ligulate, pink, sparsely glandular-puberulent at the base of stigma lobes but glabrous from the middle to the top of stigma lobes, 3.6–3.7 mm long. Capsule straight in relation to pedicel, linear, glabrous, 7.5–10 cm long, 2–2.5 mm in diameter, straight, dehiscing loculicidally to base, splitting along one suture, straight, not twisted.

##### Phenology.

Flowering occurs from March to April and fruiting from March to June.

##### Etymology.

The genus is named for the famous botanist, Dr. / Prof. Michael Möller, from the Royal Botanic Garden Edinburgh, and the species is named for Vietnam, which holds the first discovered and only known location for the species.

### Preliminary conservation status

Based on the result of our joint field surveys in the type locality and adjacent regions, the EOO and AOO of *Michaelmoelleria vietnamensis* are about 2.65 km^2^ and 0.02 km^2^, respectively. So far, only one population of this species has been recorded along the local stream on the sandstone hills of Binh Dinh province, southern Vietnam, but we believe that more *M. vietnamensis* populations will be found in the hills and mountains of southern Vietnam. If that is the case, the Extent of Occurrence (EOO) and Area of Occupancy (AOO) of this species will increase. The beautiful flowers and variegated leaves have led to its over-harvesting by local people who have sold it as an ornamental plant. For example, some local people are selling them on the digital networking platform, Facebook. This activity has caused a decline in the quantity of *M. vietnamensis*. Moreover, our field surveys showed that *M. vietnamensis* only occurred in the sandstone hills of Tay Giang community, Tay Son district, Binh Dinh province. Man-made *Eucalyptus* forests have severely fragmented the natural habitat. According to the Guidelines for Using the IUCN Red List Categories and Criteria (IUCN 2019), we access this taxon as a Critically Endangered species (CR B1+B2ab (iii, v)).

## Discussion

In March 2018, a plant having bluish-purple flowers of Gesneriaceae was collected in southern Vietnam. Neither the collectors nor the researchers on the family were able to allocate it to any known genus at that time. Collectors once thought that it might be a member of *Deinostigma* because this genus is distributed from South China to Central Vietnam (Möller et al. 2016), and shows superficial similarities to *Deinostigma* in general appearance. For example, the caulescent habit (*D. cicatricosa*, *D. cyrtocarpa*, *D. minutihamata* (D.Wood) D.J.Middleton & H.J.Atkins), usually more and less fleshy leaves (in most *Deinostigma* species except three species as mentioned above) and fleshy stems, but differs in long and zigzag narrowly infundibuliform corolla tube, four fertile stamens and two lingulate stigmas. The other morphological similar genus is *Tribounia*, a genus endemic to Thailand (Middleton and Möller 2012). The two genera share the characters of zigzag corolla tube and rounded corolla lobes but can be distinguished by the number of fertile stamens. Based on those morphological characters above, we confirmed that it does not belong to *Deinostigma* or *Tribounia*. Furthermore, after consulting the related literature (Burtt 1954, 1963; Wang et al. 1990, 1998; Li and Wang 2005; Weber et al. 2011c, 2013), we also could not find any genus in which to place this unknown species.

**Figure 5.**
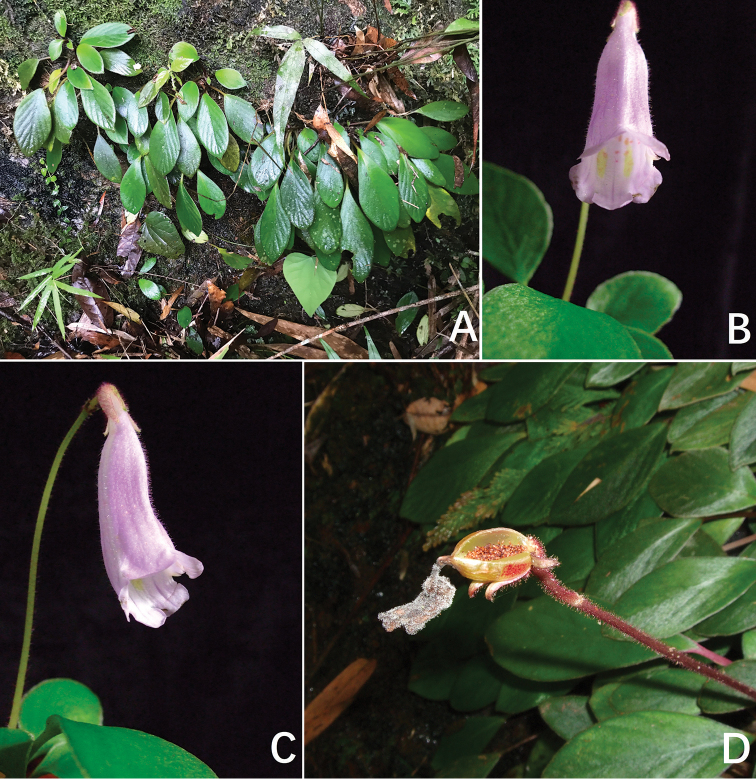
Photos of *Cathayanthe biflora* Chun, the related genus/species of *Michaelmoelleria vietnamensis* F. Wen, Z.B. Xin & T.V. Do **A** habitat **B** cyme and frontal view of flower **C** cyme and lateral view of flower **D** fruit. Photos by Fang Wen, arranged by Wen-Hua Xu.

**Figure 6.**
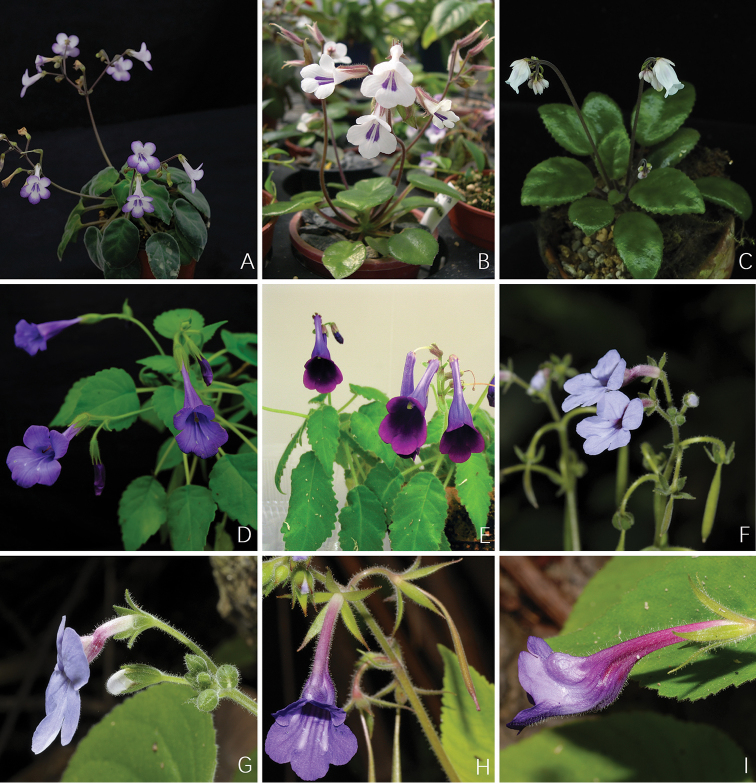
Photos of *Deinostigma* W.T.Wang & Z.Y.Li (**A–E**) and *Tribounia* D.J.Middleton (**F–I**), the morphologically similar genera and species of *Michaelmoelleria* F.Wen, Y.G.Wei & T.V.Do (*M. vietnamensis* F.Wen, Z.B.Xin & T.V.Do) **A***Deinostigma eberhardtii* (Pellegr.) D.J.Middleton & H.J.Atkins **B***D. tamiana* (B.L.Burtt) D.J.Middleton & H.J.Atkins **C***D. cycnostyla* (B.L.Burtt) D.J.Middleton & H.J.Atkins **D***D. cicatricosa* (W.T.Wang) D.J.Middleton & Mich.Möller **E***D. cyrtocarpa* (D.Fang & L.Zeng) Mich.Möller & H.J.Atkins **F–G***Tribounia venosa* (Barnett) D.J.Middleton **H–I***T. grandiflora* D.J.Middleton. **A–E** photos by Fang Wen **F–I** photos by David Middleton, arranged by Wen-Hua Xu.

The phylogenetic relationship was largely congruent with previous studies (Möller et al. 2009, 2011a, 2016; Middleton et al. 2015, 2018). *Michaelmoelleria vietnamensis* and its morphologically similar genus *Deinostigma* is recovered within a polytomy but the phylogenetic relationship of them is distant. It also shows a more distant relationship between *Michaelmoelleria vietnamensis* and the other similar genus *Tribounia*. However, our analyses of DNA sequence data suggest that *Michaelmoelleria vietnamensis* is closely related to the genus *Cathayanthe* with strong support (PP = 1, BS = 100) and both of them sister to the clade comprised of the genera of *Allocheilos*, *Gyrocheilos*, *Liebigia*, and *Didymocarpus* with strong support of BI analysis (PP = 0.99) but weak support of ML analysis (BS = 69). Morphologically, *Michaelmoelleria vietnamensis*, which represents this new genus endemic to Vietnam, can be easily distinguished from the genera of *Cathayanthe*, *Allocheilos*, *Gyrocheilos*, *Liebigia*, and *Didymocarpus* by these distinct characters: fleshly stem and leaves alternate on elongated aerial stem. In addition, only two genera’s chromosome numbers among above-mentioned genera were reported (*Liebigia speciosa* (Blume) DC. in *Liebigia*: *2n* = *28* or *32*; *Didymocarpus* ssp.: *2n* = *20*, *22*, *24*, *28*, *32*, *36*, *44*, *54*) (Möller and Pullan 2015 onwards; Yang et al. 2019). Furthermore, the chromosome numbers of *Allocheilos* W.T. Wang (two species, both endemic to China) and *Gyrocheilos* W.T. Wang (six species and two varieties, distributed from South China to North Vietnam), which are the relative genera of *Michaelmoelleria*, had never been reported before (Li and Wang 2005; Middleton 2015). The cytological evidence showed *2n = 36*, which is the difference from above genera, except *Didymocarpus pedicellatus* R.Br. (Mehra and Vasudevan 1972; Vasudevan 1976). Building on these, we treat *Michaelmoelleria vietnamensis* as a distinct genus of tribe Didymocarpeae. All distinguishing characters for identification of *Michaelmoelleria*, *Cathayanthe* (Fig. 5), *Deinostigma* (Fig. 6A–E) and *Tribounia* (Fig. 6F–I) are listed in Table 1.

**Table 1.** Comparison of morphological characters *Michaelmoelleria*, *Cathayanthe*, *Deinostigma* and *Tribounia*.

**Characters**	*** Michaelmoelleria ***	*** Cathayanthe ***	*** Deinostigma ***	*** Tribounia ***
Life form	perennial	perennial	perennial	probably annual
Stem	glabrescent to glabrous when ageing	rhizomatous, stemless	multifarious, from short and constrictive to procumbent; from glabrous to pubescent and puberulent	with hairs and often hooked at the tip
Leaves	alternate on elongate aeriald stem, 12–20 or more	few, 4–8, basal	alternate, numerous	opposite, numerous
Lea-blade	ovate to elliptic, both surfaces glabrous	oblanceolate to obovate or elliptic, adaxially whitish to brownish sericeous, abaxially appressed pubescent	multifarious, slightly peltate or not, glabrous or eglandular pubescent on both surfaces	ovate, densely pubescent adaxially and abaxially mostly with eglandular pubescents (occasional glandular pubescent present in *Tribounia grandiflora*)
Calyx	Actinomorphic, 5-parted to the base	zygomorphic, 2-lipped	divided to base, elliptic, those on ventral side slightly longer and wider	equally 5-partite almost to base
Corolla	tube narrowly curving to zigzag infundibuliform-tubular, and bent at about 90° angle in the middle of corolla tube, and gradually slightly swollen from the middle to the base of the tube; dramatically enlarged to be trumpet-shaped from the middle of the corolla tube toward the limb	tube tubular, nearly straight to slightly bent, slightly gibbous abaxially toward limb, much longer than limb	tube infundibuliform, lower lip 3-lobed, upper lip 2-lobed, lobe apices rounded	zygomorphic, of a narrow lower tube which widens into an infundibuliform upper tube which has a prominent boss on the dorsal surfacew
Fertile stamens number	4	2	2	2
Ovary	cylindric-linear, glabrous, 3.5–4 cm long	narrowly ellipsoid, ca. 6 mm long	fusform, long or short, with different indumentum, from glabrous to glandular- and eglandular-puberulent	cylindrical, densely glandular pubescent, ca. 6 mm long in *T. venosa*, 7.5–11.0 mm long in *T. grandiflora*
Stigma	2-lobed, often gathering together	subcapitate, lower part developing	ligulate, upper lip usually vestigial and only lower lip developing, broad, flat and weakly 2-lobed	capitate

## Supplementary Material

XML Treatment for Michaelmoelleria

XML Treatment for Michaelmoelleria vietnamensis

## Appendix 1

The following is a list of used samples that are ordered alphabetically by taxon with their GenBank accession number of *trnL*-*trnF* and ITS sequences respectively. The new taxa whose sequences are newly published are listed with the complete voucher information.

*Aeschynanthus rhododendron*HQ632895, HQ632993; *Aeschynanthus roseoflorus*HQ632896, FJ501333; *Agalmyla glabra*HQ632892, HQ632989; *Agalmyla paucipilosa*HQ632893, HQ632990; *Allocheilos guangxiensis*HQ632897, HQ632994; *Allostigma guangxiense*HQ632880, HQ632977; *Anna submontana*FJ501542, FJ501362; *Briggsiopsis delavayi*HQ632879, HQ632976; *Cathayanthe biflora*HQ632899, HQ632996; *Cathayanthe biflora* China: Hainan province, Shuiman Town, near Wuzhishan Mountain, WYG180606-01, 6 Jun., 2018, IBK!, MN787055, MN759631; *Chayamaritia smitinandii*KP325432, KP325425; *Codonoboea albomarginata*AJ492297, HQ632961; *Codonoboea codonion*JF912538, JF912565; *Codonoboea corrugata*FJ501484, HQ632962; *Codonoboea elata*JF912523, JF912550; *Codonoboea floribunda*JF912539, JF912566; *Codonoboea leucocodon*JF912540, JF912567; *Codonoboea malayana*JF912541, JF912568; *Codonoboea pumila*JF912543, JF912570; *Codonoboea racemosa*JF912544, JF912571; *Codonoboea venusta*JF912545, JF912572; *Conandron ramondioides*FJ501515, FJ501340; *Cyrtandra pendula*FJ501530, FJ501354; *Cyrtandra pulchella*HQ632906, EU919941; *Damrongia lacunosa*KU203896, KU203801; *Damrongia purpureolineata*KU203893, KU203798; *Deinostigma cyrtocarpa*JX506777, JX506885; Didissandra elongata ssp. minorKP325427, KP325420; *Didissandra frutescens*FJ501522, JN934793; *Didissandra* sp. KP325429, KP325422; *Didymocarpus antirrhinoides*FJ501513, DQ912671; *Didymocarpus villosus*HQ63290, HQ633001; *Didymostigma obtusum*HQ632875, HQ632971; *Didymostigma trichanthera*HQ632876, HQ632972; *Glabrella mihieri*FJ501544, FJ501363; Gyrocheilos chorisepalus var. synsepalusHQ632900, HQ632997; *Gyrocheilos lasiocalyx*HQ632901, HQ632998; *Hemiboea fangii*HQ632882, HQ632979; *Hemiboea follicularis*HQ632885, HQ632982; *Henckelia anachoreta* 1 HQ632870, HQ632966; *Henckelia bifolia*JF912522, JF912549; *Henckelia dielsii*HQ632871, HQ632967; *Henckelia floccosa*FJ501486, HQ632964; *Henckelia grandifolia*JF912527, JF912554; *Henckelia incana*HQ632869, HQ632965; *Henckelia longisepala*HQ632868, HQ632963; *Henckelia pumila* 1 JF912529, JF912556; *Henckelia pumila* 2 FJ501491, FJ501327; *Henckelia urticifolia* 1 DQ872821, DQ872835; *Henckelia urticifolia* 2 JF912532, JF912559; *Henckelia urticifolia* 3 FJ501492, FJ501328; *Hexatheca fulva*HQ632873, HQ632969; *Liebigia barbata*FJ501538, DQ912668; *Loxostigma glabrifolium*HQ632910, HQ633006; *Loxostigma griffithii*FJ501508, FJ501338; *Lysionotus pauciflorus*FJ501497, FJ501331; *Lysionotus petelotii*FJ501496, HQ632974; *Metapetrocosmea peltata*HQ632872, HQ632968; *Microchirita caliginosa*FJ501488, FJ501325; *Microchirita involucrata* 2 JF912526, JF912553; *Microchirita mollissima*JF912528, JF912555; *Microchirita sericea*JF912521, JF912548; *Microchirita tubulosa*JF912531, JF912558; *Microchirita viola*JF912533, JF912560; *Middletonia regularis*KU203884, KU203789; *Oreocharis dasyantha*HQ632918, HQ633014; *Oreocharis jiangxiensis*HQ632933, HQ633029; *Michaelmoelleria vietnamensis* VIETNAM. Binh Dinh province, Tay Son district, Tay Giang community, La stream. 13°55'59"N, 108°45'43"E, ca. 148 m, 29 Mar., 2018, WYG180329-01, IBK!, MN787054, MN783373; *Oreocharis urceolata*HQ632922, HQ633018; *Ornithoboea flexuosa*KU203931, KU203836; *Paraboea clarkei*JN934715, JN934757; *Petrocodon ainsliifolius*HQ632941, HQ633038; *Petrocodon dealbatus*FJ501537, FJ501358; *Petrocodon scopulorum*HQ632947, HQ633044; *Petrocosmea kerrii*FJ501502, FJ501334; *Petrocosmea nervosa*AJ492299, FJ501335; *Primulina gemella*FJ501523, FJ501345; *Primulina luochengensis*HQ632949, HQ633046; *Primulina tabacum*AJ492300, FJ501352; *Pseudochirita guangxiensis*HQ632908, HQ633003; *Raphiocarpus sinicus*HQ632877, HQ632973; *Ridleyandra petiolata*HQ632935, HQ633032; *Ridleyandra quercifolia*HQ632936, HQ633033; *Somrania albiflora*KU203887, KU203792; *Spelaeanthus chinii*FJ501457, FJ501307; *Streptocarpus glandulosissimus*KR703972, AF316918; *Streptocarpus rexii*AJ492305, AF316979; *Tribounia grandiflora*JX839281, JX839280; *Tribounia venosa*JX839282, JX839283.
